# Origin and higher-level diversification of acariform mites – evidence from nuclear ribosomal genes, extensive taxon sampling, and secondary structure alignment

**DOI:** 10.1186/s12862-015-0458-2

**Published:** 2015-09-02

**Authors:** A R Pepato, P B Klimov

**Affiliations:** Departamento de Zoologia, Instituto de Ciências Biológicas, Universidade Federal de Minas Gerais, Av. Antonio Carlos, 6627, 31270-901 Belo Horizonte, Brazil; Department of Ecology and Evolutionary Biology, University of Michigan, 1109 Geddes Ave, Ann Arbor, MI 48109-1079 USA; Tyumen State University, 10 Semakova St, Tyumen, 625003 Russia

## Abstract

**Background:**

Acariformes is the most species-rich and morphologically diverse radiation of chelicerate arthropods, known from the oldest terrestrial ecosystems. It is also a key lineage in understanding the evolution of this group, with the most vexing question whether mites, or Acari (Parasitiformes and Acariformes) is monophyletic. Previous molecular studies recovered Acari either as monophyletic or non-monophyletic, albeit with a limited taxon sampling. Similarly, relationships between basal acariform groups (include little-known, deep-soil 'endeostigmatan' mites) and major lineages of Acariformes (Sarcoptiformes, Prostigmata) are virtually unknown. We infer phylogeny of chelicerate arthropods, using a large and representative dataset, comprising all main in- and outgroups (228 taxa). Basal diversity of Acariformes is particularly well sampled. With this dataset, we conduct a series of phylogenetically explicit tests of chelicerate and acariform relationships and present a phylogenetic framework for internal relationships of acariform mites.

**Results:**

Our molecular data strongly support a diphyletic Acari, with Acariformes as the sister group to Solifugae (*PP* =1.0; BP = 100), the so called Poecilophysidea. Among Acariformes, some representatives of the basal group Endeostigmata (mainly deep-soil mites) were recovered as sister-groups to the remaining Acariformes (i. e., Trombidiformes + and most of Sarcoptiformes). Desmonomatan oribatid mites (soil and litter mites) were recovered as the monophyletic sister group of Astigmata (e. g., stored product mites, house dust mites, mange mites, feather and fur mites). Trombidiformes (Sphaerolichida + Prostigmata) is strongly supported (*PP* =1.0; *BP* = 98–100). Labidostommatina was inferred as the basal lineage of Prostigmata. Eleutherengona (e. g., spider mites) and Parasitengona (e. g., chiggers, fresh water mites) were recovered as monophyletic. By contrast, Eupodina (e. g., snout mites and relatives) was not. Marine mites (Halacaridae) were traditionally regarded as the sister-group to Bdelloidea (Eupodina), but our analyses show their close relationships to Parasitengona.

**Conclusions:**

Non-trivial relationships recovered by our analyses with high support (*i.e*., basal arrangement of endeostigmatid lineages, the position of marine mites, polyphyly of Eupodina) had been  proposed by previous underappreciated morphological studies. Thus, we update currently the accepted taxonomic classification to reflect these results: the superfamily Halacaroidea Murray, 1877 is moved from the infraorder Eupodina Krantz, 1978 to Anystina van der Hammen, 1972; and the subfamily Erythracarinae Oudemans, 1936 (formerly in Anystidae Oudemans, 1902) is elevated to family rank, Erythracaridae stat. ressur., leaving Anystidae only with the nominal subfamily. Our study also shows that a clade comprising early derivative Endeostigmata (Alycidae, Nanorchestidae, Nematalycidae, and maybe Alicorhagiidae) should be treated as a taxon with the same rank as Sarcoptiformes and Trombidiformes, and the scope of the superfamily Bdelloidea should  be changed. Before turning those findings into nomenclatural changes, however, we consider that our study calls for (i) finding shared apomorphies of the early derivative Endeostigmata clade and the clade including the remaining Acariformes; (ii) a well-supported hypothesis  for Alicorhagiidae placement; (iii) sampling the families Proterorhagiidae, Proteonematalycidae and Grandjeanicidae not yet included in molecular analyses; (iv) undertake a denser sampling of clades traditionally placed in Eupodina, Anystina (Trombidiformes) and Palaeosomata (Sarcoptiformes), since consensus networks and *Internode certainty* (IC) and *IC All* (ICA) indices indicate high levels of conflict in these tree regions. Our study shows that regions of ambiguous alignment may provide useful phylogenetic signal when secondary structure information is used to guide the alignment procedure and provides an R implementation to the Bayesian Relative Rates test.

**Electronic supplementary material:**

The online version of this article (doi:10.1186/s12862-015-0458-2) contains supplementary material, which is available to authorized users.

## Background

Mites, a ubiquitous and megadiverse group of chelicerates, are traditionally classified into two large assemblages: Acariformes (= Actinotrichida) and Parasitiformes (= Anactinotrichida), as reviewed by [1] and references therein. Recent analyses employing partial ribosomal and mitochondrial markers [2, 3], combined partial ribosomal genes and morphology [4], EST data sets [5] and genomic scale datasets [6, 7] suggest that Acari is a diphyletic group, something that contrasts with earlier studies employing partial ribosomal genes alone [8] or combined with morphology [9, 10]. Specifically, studies that recovered mites as a diphyletic group (except for [7]) proposed an unranked lineage, Acariformes + Solifugae, for which Pepato et al. [4] adopted the name Poecilophysidea.

Ingroup relationships of Acariformes were proposed based on morphology [11, 12] as follows: many basal Endeostigmata and all Oribatida (including Astigmata) form the Sarcoptiformes Reuter, 1909 (morphologically defined mainly by the presence of rutella); in contrast, another large radiation, the Trombidiformes Reuter, 1909, is represented by Sphaerolichida OConnor, 1984 and the megadiverse Prostigmata Kramer, 1877 (defined mainly by the lack of rutella and reduction of dorsal setation). Prostigmata were hypothesized to include three main groups: Labidostommatina Krantz, 1978 plus Eupodina Krantz, 1978, Anystina van der Hammen, 1972 and Eleutherengona Oudemans, 1909 [11, 13, 14], although Eupodina was placed either as sister group of Anystina [11] or as the basal lineage of the remaining Prostigmata [13]. Unfortunately, these hypotheses were not fully tested with molecular data.

Here, we used nearly complete sequences of the small and large subunits of nuclear rDNA (SSU and LSU) of all major lineages of acariform mites and numerous chelicerate and non-chelicerate outgroups to infer chelicerate relationships, test the hypotheses on the placement of Acariformes, and their internal relationships. Recently published studies on the Acariformes internal phylogeny put relatively little emphasis on outgroup sampling, taking a monophyletic Acari as granted and including just a few Parasitiformes terminals [15, 16], whereas those presenting broader taxon sampling [2, 4] missed crucial acariform families such as Lordalychidae and deep soil Endeostigmata (never sequenced previously, except for *Alicorhagia*) and were based on SSU and small stretches of the LSU gene (~300 bp); the only published study on Chelicerata using large portions of the LSU gene [3] has a biased in-group Acariformes sampling and did not attempt to employ the secondary structure in order to align sequences and improve phylogenetic analyses [17, 18]. Our study accounts for these shortcomings, offering an opportunity to investigate questions related to the origin of Acariformes with a greater accuracy.

## Results

### Data set characteristics

The length of SSU rDNA sequences (helices H47 to H1399) of the 228 species used in this study ranged from 1691 bp in *Macrocheles* (Parasitiformes, Mesostigmata) to 2429 bp in *Peripatus* (Onychophora). Among the Chelicerata, the longest sequence (2135 bp) was observed in *Haplochthonius* (Acariformes, Oribatida). For LSU rDNA (helices H224 to H2675), sequences ranged from 3204 bp in *Amblyseius* (Parasitiformes, Mesostigmata) to 5367 bp in *Penaeus* (Decapoda). Among the Chelicerata, the longest sequence was found in *Brevipalpus* (Acariformes, Trombidiformes) with at least 3989 bp (a short region at the 3’ end was not sequenced for this species). The two genes were aligned using a secondary structure consensus approach (see below). The final aligned matrix (after exclusion of regions of ambiguous alignment, RAAs) contained 4503 nt positions; of them, 1240 were constant, and 2421 were parsimony informative. There were 2251 paired (the number is uneven because a small stretch of pairing region was not sequenced) and 2252 non-paired nucleotide positions in the alignment.

**Fig. 1:**
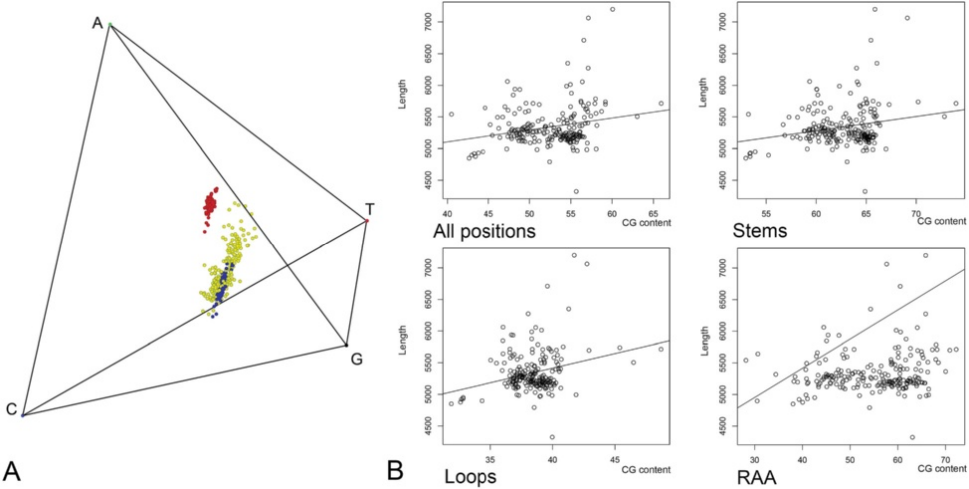
Nucleotide composition. **a**- Tetrahedric graph showing nucleotide composition of loop (red), stem (blue), and regions of ambiguous alignment (RAAs, yellow) of rDNA. **b**- Nucleotide composition (CG content) plotted against the sequence length for the entire rDNA alignment and the three partitions; regression line is given for each graph

We detected a strong bias toward adenines in the loop regions (colored by red on Fig. 1a) relative to stems (blue); values for regions of ambiguous alignment (yellow) overlapped with those for stems but were more spread. The sequence length and GC content were positively correlated (*ρ* = 0.23, *P* < 0.01, Fig. 1b), similarly to that reported previously [17]. A weak positive correlation (*ρ* = 0.15, *P* = 0.0337) was detected for RAA; these regions account for most of the observed length variation. The observed differences in nucleotide frequency among secondary structure defined partitions suggest separate models of molecular evolution; irrespective of modeling covarying sites in stem regions (see the Methods and Discussion sections below). From the 228 taxa, 116 deviated from the maximum likelihood stationary composition. Despite this, arguably spurious groupings due to base composition were not detected in the phylogenetic analyses (see the following section).

**Fig. 2:**
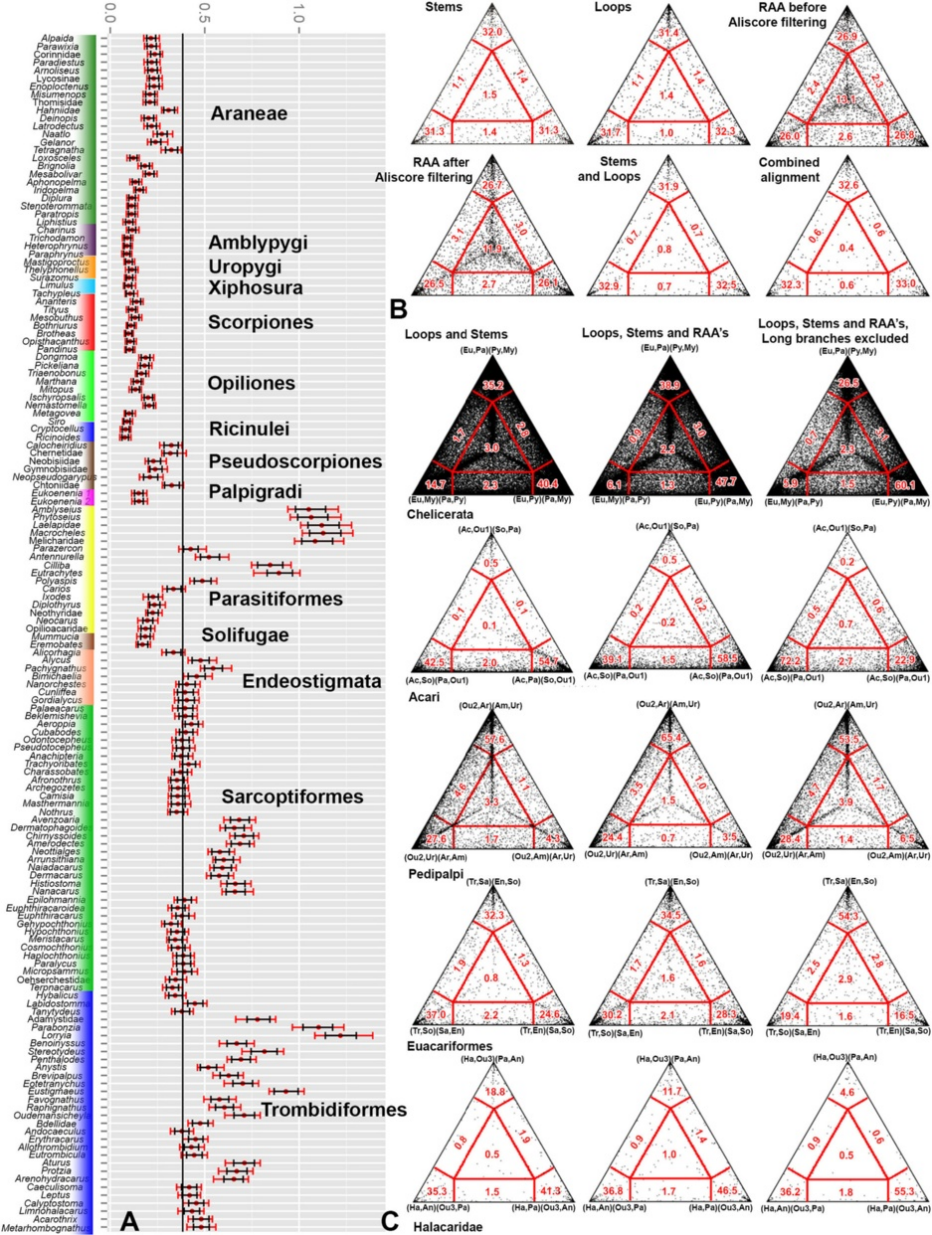
Bayesian Relative Rates test and Likelihood Mapping. **a**- Bayesian Relative Rates of substitutions. For each horizontal bar, the mean rate (red dot), the credible interval (black) and the range (red) are given. **b**- Likelihood mapping for stems, loops, RAA partitions before and after *Aliscore* filtering, combined stems and loops, and combined stems, loops and filtered RAAs. **c**- Likelihood mapping for selected bipartitions. The mapping was performed for concatenated stems and loops, all partitions, and all partitions with rapidly evolving lineages (long branches) excluded. Abbreviations. Chelicerata: Eu, Euchelicerata; My, Myriapoda; Py, Pycnogonida; Pa, Pancrustacea. Acari: Ac, Acariformes: So, Solifugae; Pa, Parasitiformes; Ou1, outgroup Euchelicerata. Pedipalpi: Ou2, Outgroup Euchelicerata; Ur, Uropygi; Ar, Araneae; Am, Amblypygi. Euacariformes: Tr, Trombidiformes; So, Solifugae; Sa, Sarcoptiformes; En, Endeostigmata. Ha, Halacaridae; An, Caeculidae + Erythracarinae; Pa, Parasitengona; Ou3, all other Acariformes

**Fig. 3:**
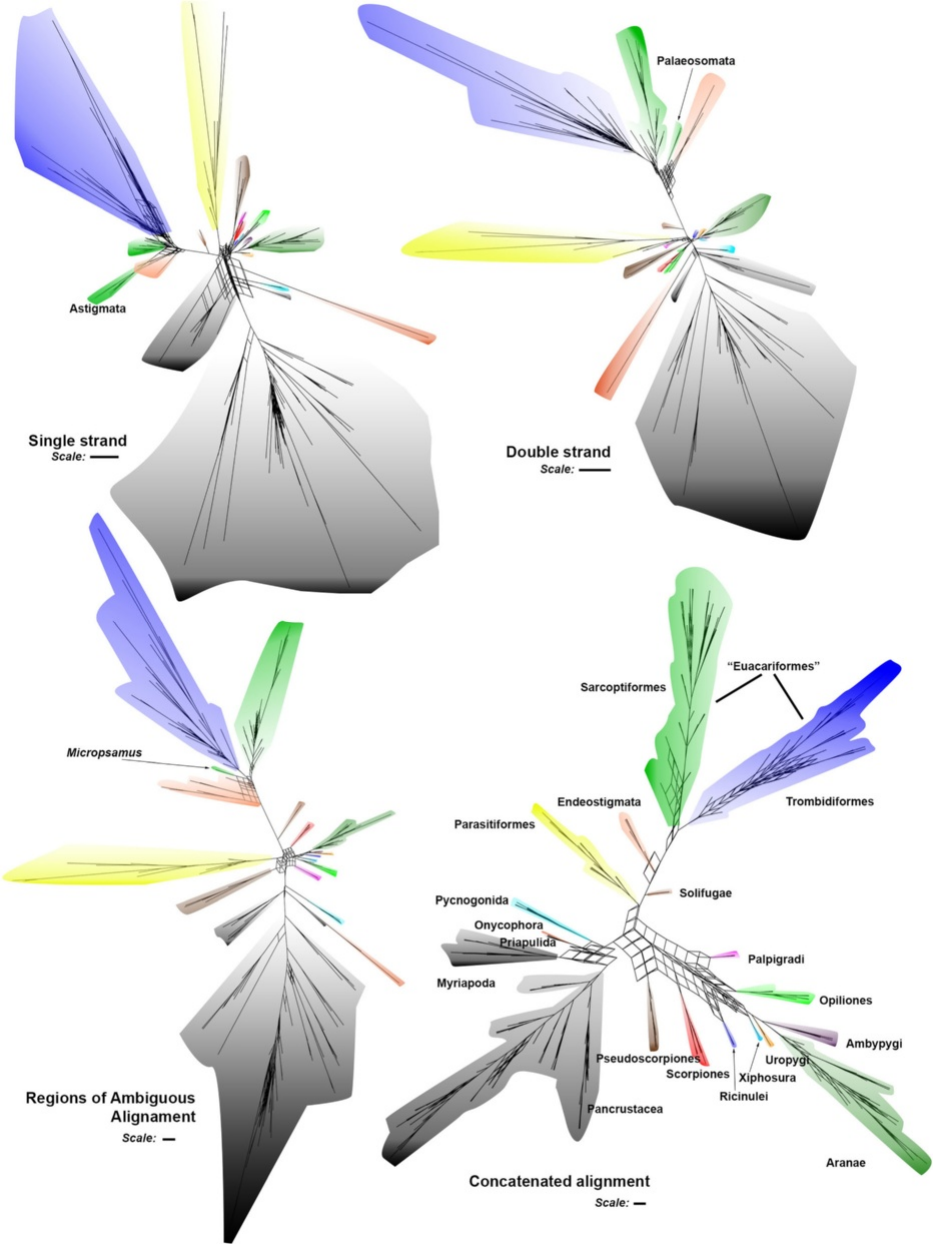
Consensus networks. Each consensus network was calculated from 1000 bootstrap trees (with bipartitions with frequency under 0.1 excluded). Taxon color codes match those on Fig. 2a. The network from the RAA alignment was produced after *Aliscore* filtering. Scale bars: 0.1 nucleotide substitutions per site

To evaluate the saturation level in our dataset, we calculated the index of substitution saturation (Iss), a critical value of the parameter at which a phylogenetic analysis will begin to fail to recover the true tree for either symmetrical (Iss.c sym) or asymmetrical (Iss.c asym) topology. If Iss of a particular dataset is substantially smaller than both Iss.c sym and Iss.c asym, no significant saturation is presumed [19, 20]. Our analysis suggests that none of the secondary structure defined partitions are saturated – Iss, Iss.C Sym, and Iss C Asym were as follows (ambiguous positions excluded): Loops 0.159, 0.700, and 0.395; Stems 0.218, 0.686, and 0.366; and RAA after filtered using *Aliscore* 0.640, 11.936, 19.025. Likelihood mapping (LM) and consensus networks (CN) analyses conducted on bootstrap trees displayed contrasting results with regard to the phylogenetic signal in individual partitions and combined alignment. The LM analysis (Fig. 2b) indicated that the stem and loop partitions are similarly informative, leaving small fractions of quartets in the star-like tree region (1.5 and 1.4 %, respectively), and RAA regions with a larger proportion of unsolved quartets irrespective of being analyzed before or after *Aliscore* filtering (11.9 and 13.1 %, respectively). The CN analysis (Fig. 3) showed fewer conflicting bipartitions and a resolution near to that observed for the RAA concatenated tree. It is interesting that a major acariform lineage, Astigmata, was inferred basal at the Acariformes tree when the loop partition alone was analyzed. This position was recovered previously using unpartitioned analysis of the 18S gene [21], but is considered spurious based on morphology (*i.e*., the presence of the opisthonotal glands suggests its origin within derived Oribatida). Similar warnings concerning the loop regions were issued previously, although they were linked to high levels of homoplasy detected by saturation tests [22], something that was not observed here (see above). In fact, tree certainty for the loop partition (Tree certainty including all conflicting bipartitions (TCA) for this tree: 90.4, Relative TCA for the best loop ML tree: 0.40) is considerably lower than that for stem partition (respectively 107.0 and 0.48), hence our analysis highlights potential non-phylogenetic signal in the loop regions, which may solve quartets in LM analyses but may introduce conflicts in the topology. These results indicate the need for careful evaluation of trees based on the total evidence approach because they can be influenced by a single rDNA partition.

### Molecular phylogenetic analyses

**Fig. 4:**
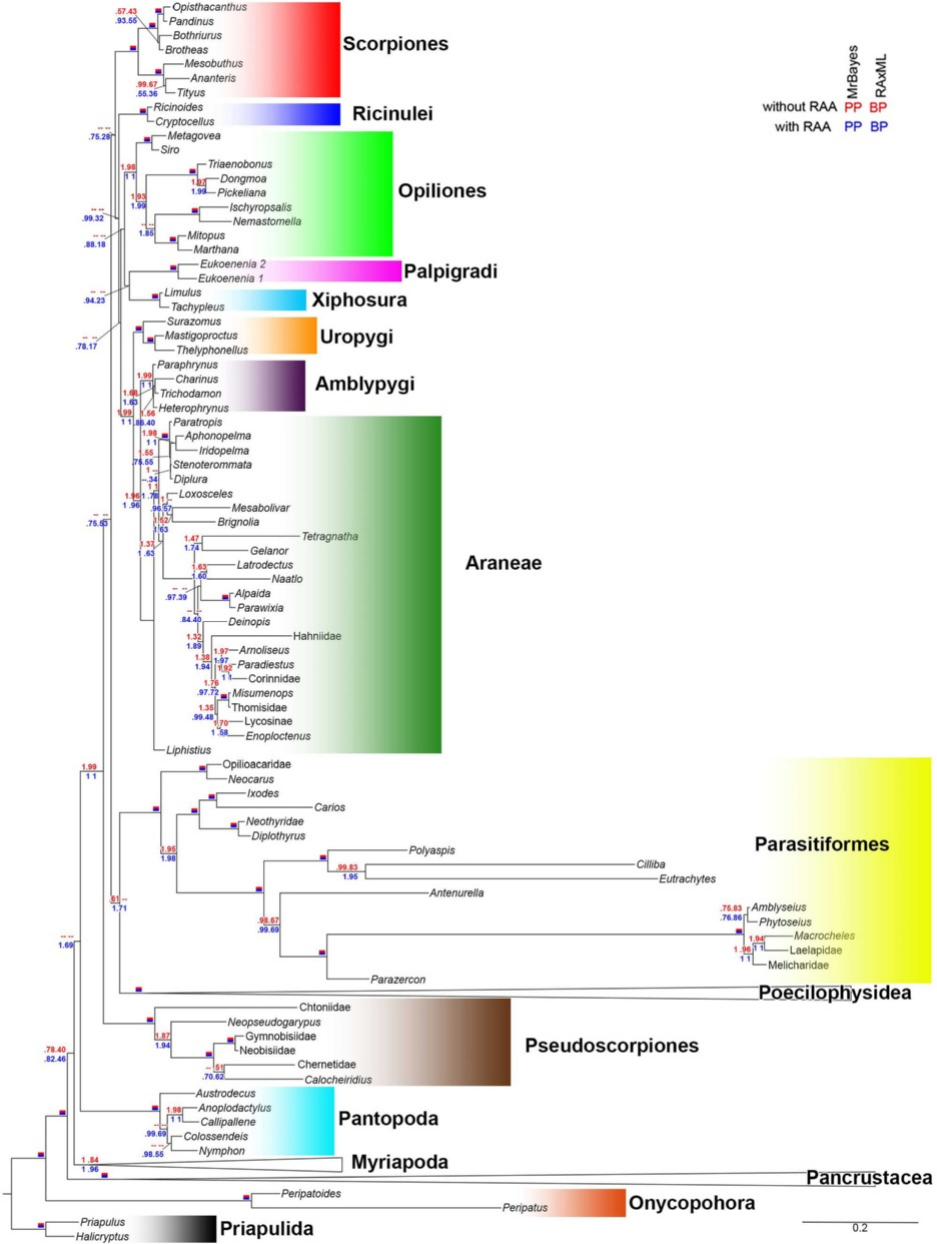
Relationships between chelicerate orders. Topology inferred by the partitioned (stems, loops and RAAs) ML analysis. For each node, posterior probabilities and bootstrap support values are given for three (blue) and two (red) partition analyses. If all values are 100 %, the node is marked with a solid red and blue rectangle. Dashes indicate internodes not recovered in all analyses

**Fig. 5:**
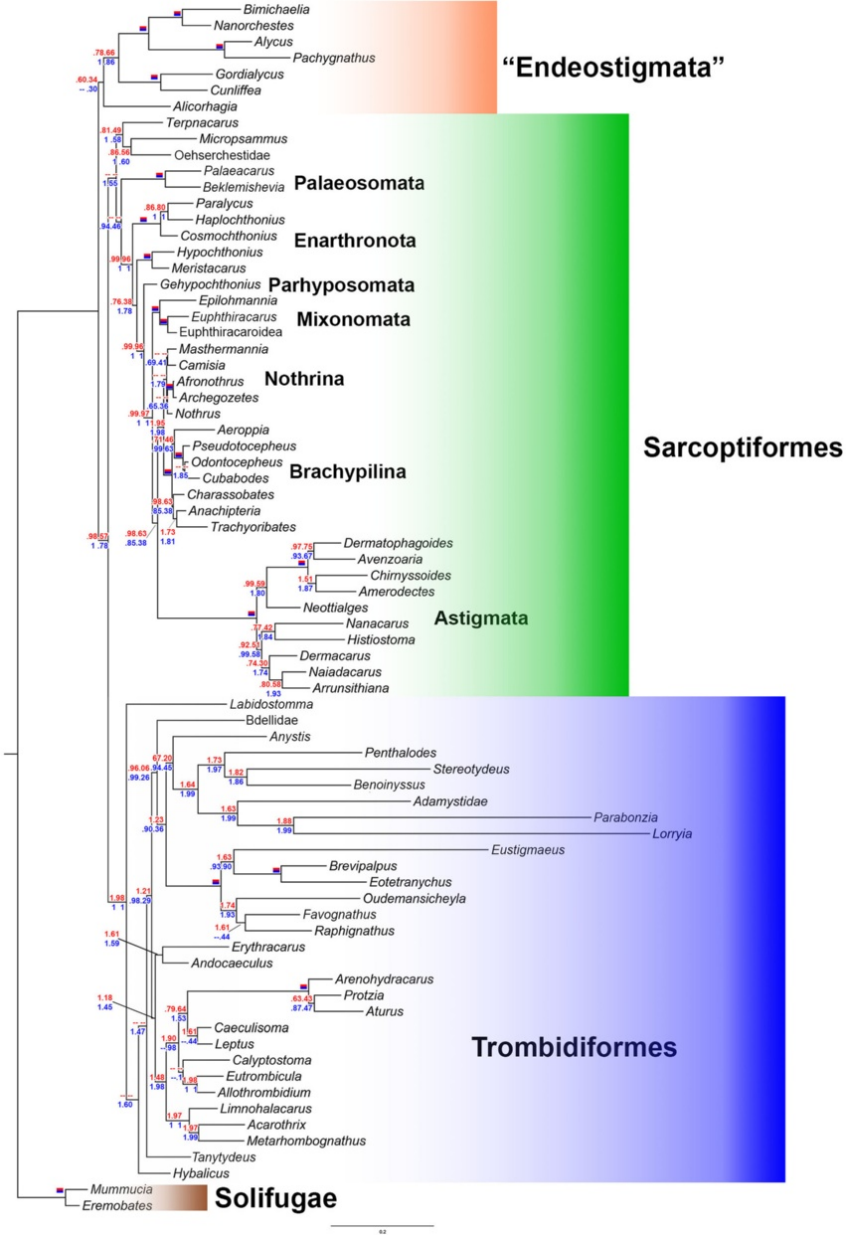
Relationships of Poecilophysidea (Acariformes and Solifugae). Maximum likelihood tree, partitioned analysis (stems, loops and RAAs). See the Fig. 4 caption for conventions in showing node support values

Bayesian and Maximum Likelihood trees inferred from the two-partition (stems, loops) and three-partition (stems, loops, RAAs) datasets were largely congruent (Figs. 4 and 5, Additional file 4). In order to test for potential Long Branch Attraction Artifact (LBA), terminals for which the Bayesian Rates test showed no overlap of their credible intervals to those of the remaining Acariformes (Fig. 2a) were removed. Then this dataset was partitioned (stems, loops, RAA) and re-analyzed. This reduced dataset was also subjected to a likelihood analysis mapping for some alternative resolutions of relevant bipartitions (Fig. 2c). No grouping due to putative LBA could be detected in this way.

### Arthropod relationships

Most of the well-supported outgroup relationships were similar to previous molecular studies, indicating the presence of useful, phylogenetically informative signal in our target genes. Pancrustacea was recovered with a very strong support (100 % of BP and PP in all analyses), whereas Myriochelata (Myriapoda plus Chelicerata) was recovered with a low support in both analyses (two partitions, Bayesian and ML: *PP* = 78, *BP* = 40; three partitions, Bayesian and ML: *PP* = 82, *BP* = 46). Chelicerata in its traditional sense (Pycnogonida + Euchelicerata) was recovered only after the inclusion of the RAA data (*PP* = 100, *BP* = 69). We also observed an increasing proportion of quartets ((Pancrustacea, Myriapoda)(Chelicerata, Pycnogonida)) from the pre-aligned, three-partition, and long branch pruned dataset (Fig. 2c).

Two alternative hypotheses to Myriochelata, Cormogonida (= Euchelicerata (Myriapoda("Crustacea",Hexapoda))) [23, 24] and Mandibulata (= Myriapoda ("Crustacea", Hexapoda)) [25–27], could not be rejected statistically by the AU test given our data (Table 1). Euchelicerata was recovered under all analytical approaches and datasets, with a high support.

**Table 1:** Hypothesis testing using AU statistics

Hypothesis	Without RAAs		With RAAs	
	δLnl	AU	δLnl	AU
Best tree without RAA	--	0.768	**82.6**	**0.027**
Best tree with RAA	43.3	0.135	--	0.819
Cormogonida	11.3	0.518	12.3	0.358
Mandibulata	18.7	0.353	5.5	0.724
Arachnida	22.2	0.263	27.5	0.219
Pedipalpi	39.8	0.104	**145.9**	**0.002**
Haplocnemata	**66.3**	**5e-005**	**72.8**	**0.004**
Dromopoda	**68.9**	**0.014**	**86.8**	**3e-004**
Dromopoda (Solifugae excluded)	9.3	0.622	17.1	0.403
Monophyletic Acari	34.5	0.074	**43.6**	**0.038**
Acaromorpha	**56.4**	**0.003**	**79.8**	**0.001**
Parasitiformes + Opiliones	14.7	0.485	29.7	0.127
Parasitiformes + Pseudoscorpiones	2.0	0.729	13.9	0.392
Cryptognomae	14.6	0.430	33.6	0.173
Non-Euacariformes	60.9	0.124	11.6	0.379
Sarcoptiformes	14.7	0.434	13.0	0.471
Eupodina	**74.6**	**0.007**	**130.2**	**3e-004**
Bdelloidea	37.5	0.208	**46.3**	**0.041**
Anystidae	39.1	0.217	35.7	0.208
Anystina	37.2	0.062	**51.5**	**0.009**

*P*-values below *P* < 0.05 (bold) indicate that a particular hypothesis can be statistically rejected.

### Euchelicerata relationships and monophyly of Acari

All chelicerate orders were well supported. In contrast, the class Arachnida was not recovered, although the AU test could not rule out this grouping. This situation is due to the unstable position of Xiphosura and most other Euchelicerata. Only two supra-ordinal groupings, Tetrapulmonata and Poecilophysidea (*i.e*. Acariformes + Solifugae) are stable and received substantial support.

Tetrapulmonata is a well-defined and uncontroversial clade, whereby the topology recovered here was (Uropygi (Amblypygi + Araneae)), the so-called Labellata [28] rather than the Pedipalpi clade (Amblypygi + Uropygi) typically found in morphological and combined morphological + DNA analyses [4, 9, 10, 29]. Pedipalpi was rejected by the data after including the RAA partition (Table 1).

The best supported topology did not recover mites as a monophylum. Parasitiformes is unstable, since it was found associated with Palpigradi in the ML analyses including only stems and loops (*BP* = 32) or Poecilophysidea (*PP* = 1.0, *BP* = 71) after including RAAs. Regardless, the AU test cannot reject most of alternative placements, such as a sister-group relationship with Pseudoscorpiones [2], Opiliones [6], Ricinulei (the so called Cryptognomae [30, 31]) (Table 1). Our data, however, reject Acari [32] and Acaromorpha (Acari + Ricinulei) [9, 10, 28, 29, 32], as well as alternative placements of Solifugae, such as the Haplocnemata (= Solifugae + Pseudoscorpiones [9, 10, 28, 29]) and Dromopoda (Haplocnemata + Scorpiones + Opiliones [9, 10, 29]).

### Higher-level relationships of Acariformes

Our molecular tree of the Acariformes disagrees with influential, morphology-based ideas on their systematics [14]. In our topology, the earliest basal divergence of Acariformes was “Endeostigmata” (Fig. 5), which has been considered as basal Sarcoptiformes based on morphological evidence [12]. We recovered “Endeostigmata” as a monophyletic sister group to the remaining Acariformes (a grouping referred hereafter to as “Euacariformes” for sake of simplicity) in all analyses, except for the stem and loop Bayesian analysis, which could not resolve the position of *Alicorhagia*. The non-endeostigmatan acariform clade received moderate (two partitions: *PP* = 98, *BP* = 57) to high support (three partitions: *PP* = 100, *BP* = 78). When applying a negative constraint against “Euacariformes”, neither the most probable tree sampled by Markov chain, nor the majority rule consensus tree recovered Sarcoptiformes (most of Endeostigmata + Oribatida + Astigmata), but a topology with most of Endeostigmata grouped with Trombidiformes. Sarcoptiformes sensu [12] could not be rejected in the AU test, however.

Except for Paleosomata, the remaining sarcoptiform backbone tree is well supported, especially after including RAAs, and roughly congruent with the relationships inferred from morphology: (Palaeosomata, (Enarthronota, (Parhypsomata, (Mixonomata, (Astigmata, (Brachypilina, Nothrina)))))). Here we note a major improvement in our tree over previously published DNA-based phylogenies with respect to the position of Parhyposomata; the latter is now consistent with morphological evidence (i. e., the presence of the opisthonotal glands). The hypothesis of a paraphyletic Desmonomata with respect to Astigmata remains to be tested since a key taxon, Malaconothroidea, was not included.

Trombidiformes (Sphaerolichida plus Prostigmata) was recovered with high support (*PP* = 100, *BP* = 98–100). The trombidiform tree is unstable in the basal portion, depending on whether or not RAAs were included. Regardless of different analyses, Labidostommatina comes close to the trombidiform root. Among the major divisions of Prostigmata, Eleutherengona was recovered with a high support (*PP* = 100, *BP* = 100); Eupodina was not recovered, Halacaridae (marine mites) was placed as the sister group of Parasitengona, receiving 100 % of PP in all Bayesian analyses and moderate to high support in ML analyses (*BP* = 48–89); Adamystidae and Anystinae, usually classified in Anystina, were found nested within several Eupodina; and Bdellidae was recovered as basal Prostigmata. Neither the family Anystidae nor the superfamily Bdelloidea was recovered as a monophyletic group. The best tree recovered after constraining monophyletic Eupodina was rejected by the AU test. Hypotheses obtained after constraining the clade Caeculidae+ Erythracaridae+ Parasitengona (*i.e*., excluding the sister group relationship between Halacaridae and Parasitengona), and Bdelloidea (Cunaxidae + Bdellidae) were rejected by the analyses including RAAs. The tree obtained after constraining monophyletic Anystidae could not be rejected.

### Accessing the phylogenetic signal: likelihood mapping, consensus networks, and internode certainty

To facilitate visual comparison among alternative topologies after including RAAs and excluding the fast evolving lineages detected through the Bayesian Relative Rates test (Fig. 2a), we employed Likelihood Mapping (Fig. 2c). In three cases the proportion of quartets resolved as the preferred hypothesis (*i.e*. the three-partition analysis) increases after including RAA and excluding rapidly evolving lineages: Chelicerata (=Pycnogonida + Euchelicerata) – 40.4, 47.7, and 60.1 %, respectively); “Euacariformes” (32.3 %, 34.5 %, 54.3 %); and the placement of Halacaridae as sister group of Parasitengona (41.3 %, 46.5 %, 55.3 %). The other two hypotheses Labellata (=Amblypigi + Araneae) and Poecilophysidea (=Acariformes + Solifugae) have a slight decrease in support when RAAs are included and present a slight (Labellata, 27.6 %, 24.4 %, 28.4 %) or great (Poecilophysidea, 42.5 %, 39.1 %, 72.2 %) improvement in support after exclusion of long branches.

Consensus networks based on bootstrap trees detected similar regions of conflict for stems and RAAs (Fig. 3), i.e. the basal portion of the tree; the relationships among Euchelicerate orders (except Tetrapulmonata and Poecilophysidea); and Endeostigmata relative to basal “Euacariformes”, especially Sarcoptiformes. The consensus networks from the analyses of the loop partition show the poorest resolution, especially for the basal splits (relative to Pycnogonida, Myriapoda, Pancrustacea), Euchelicerata, and for Trombidiformes relative to Sarcoptiformes. Each partition had a representative of taxa otherwise recovered as “Sarcoptiformes” misplaced closer to “Endeostigmata”. For loops it was Astigmata, for stems it was Palaeosomata, and for RAAs it was the genus *Micropsammus*.

The consensus network based on the combined dataset shows no conflicting bipartitions between “Endeostigmata” and “Euacariformes”. The “Sarcoptiformes” network contrasts to the reticulate structure of the Trombidiformes consensus network by being much more tree-like.

Numerical results for the Internode Certainty (IC) and Internode Certainty All (ICA) match perfectly the consensus networks in detecting the most conflicting regions of the three and show a gain in resolution after including RAAs (Additional file 4).

## Discussion

### Strengths and limitations

The inclusion of a large outgroup sample allowed us to compare the performance of our analyses to those of published studies utilizing single-gene, multi-gene or genomic data. The topology presented here for major arthropod lineages —(Pancrustacea, (Myriapoda, Pycnogonida, Euchelicerata)), the so-called Myriochelata or Paradoxosopoda —is commonly recovered by ribosomal gene analyses [33], although support for the alternative hypothesis, Mandibulata, was obtained from combined analyses of morphology and microRNAs [27 and references therein].

The Pancrustacea topology (Additional file 4) is largely congruent to that presented previously [34], sharing its limits and strengths. Copepods are reported as sister-group of Hexapoda with a high support (*PP* = 100, *BP* = 86–98), a controversial result also found when non-stationary models were applied to ribosomal sequences [35]. No putative morphological apomorphy can be found supporting this clade, nor was it recovered in multi-locus analyses [36, 37]. On the other hand, similarly to genomic studies, our results agree in recovering the endognath hexapods Nonoculata (Protura + Diplura), Oligostraca (Branchiura + Ostracoda + Mystacocarida), and the close relationship between barnacles and Malacostraca [37].

For relationships among the orders of Euchelicerata, we found no resolution, except for Tetrapulmonata and Poecilophysidea [2–6]. Recent genomic-scale studies [5, 7] also failed in reaching a well-supported hypothesis on ordinal relationships. They do support, however a distinct solution for in-group Tetrapulmonata, with Pedipalpi instead of Labellata, and only one study [5] recovered Poecilophysidea with moderate support. The most surprising result for intraordinal relationships was the recovery of Palpatores (Eupnoi + Dyspnoi) only after inclusion of the RAA dataset, a clade recovered in combined analyses including fossils and genomic scale datasets [38].

Phylogenetic placement of parasitiform mites remains extremely unstable. Parasitiformes was recovered as a basal lineage among Euchelicerata [3, 4, 7], as the sister group of Pseudoscorpiones [2], or in a group including Opiliones and Tetrapulmonata [6]. All of these phylogenies suggest a large gap in the fossil record for Parasitiformes, since the oldest parasitiform mites, ticks and Opilioacarida, were discovered in Burmese amber from the Cretaceous (ca. 100 Ma) [39–42]. By contrast, acariform mites are known from the early Devonian (ca. 410 Ma) Rhynie Chert of Scotland [43, 44], and Paleozoic fossils have been discovered for most of the arachnid orders [45, 46]. The traditional morphology-based groupings, Acari and Acaromorpha (Acari + Ricinulei), were statistically rejected by the AU test employing the three-partition dataset (Table 1). Furthermore, alternative placements of Solifugae also could be rejected.

### Multiple alignments of ribosomal gene sequences

In contrast to introns or coding regions, alignment of ribosomal DNA, to be biologically sensible, should account for secondary structure information (*i.e*., mature ribosomal RNA folded to form stems and loops maintained both by paired RNA regions and ribosomal proteins). Non-conserved RAA regions of rDNA are especially challenging for alignment because both secondary rDNA structures and sequences evolve in these regions. To address this, two alternative analytical approaches, consensus and individual secondary structure based alignments were developed. As part of initial exploratory analyses we tried both methods, but only the former approach is further reported here. In this method, the rationale for homology assessment is accounting for the fact that secondary structure is functionally constrained in ribosomal RNA. This method, however, usually leaves large stretches where the secondary structure is not conserved (Regions of Ambiguous Alignment, RAAs) out.

Congruence of the secondary structure consensus analyses with previous studies, the better resolution of phylogenetic trees, and bootstrap consensus networks with filtered RAAs all indicate that it is possible to take advantage of RAA regions if two conditions are met. First, based on existing eukaryotic secondary structure rDNA models, conserved regions and RAAs should be identified (bracketed), and then each RAA should be independently aligned using an automatic algorithm. It was already noted [4] that if two variable in length regions are separated by very short stretches of conserved secondary structure of sequence, most algorithms fail to recover those conserved regions. Second, accurate guide trees and realistic cost regimes should be employed. All automatic alignment methods rely on a guide tree to order pairwise alignments and a cost regime to produce scores for comparing alternative alignments. In this study, we employed trees from the 95 % credible interval of trees sampled from the posterior for each RAA to avoid biasing our output alignments to a single topology (see the Methods section below). For gap opening and extension costs, any choice is arbitrary since it is not data driven [47]. In a study on direct optimization accuracy, Liu et al. [48] observed that it performed best under a cost regime penalizing four times the gap opening relative to gap extension and substitution. In any case, the use of a single cost regime for all RAAs chosen without regard for data is far from ideal; hence the alignment was masked using a program that identifies random similarity within multiple sequence alignments based on Monte Carlo resampling within a sliding window [49].

Another method of multiple rDNA alignment is the individual secondary structure approach. In current literature this framework has been tailored to the internal transcribed spacer (ITS2), a region that is spliced out from mature rRNA. This method typically includes a pipeline starting with the *ITS2 database* (source of sequence–structure data), *foursale* or *4SALE* (alignment) and *ProfDistS3* (distance method for phylogenetic reconstruction) or a custom substitution matrix to be implemented in a maximum likelihood environment [50, 51]. This pipeline was extensively applied to ITS2 [52–54] or rarely to other markers, like mitochondrial 16S [55]. The main issue precluding a large scale application of this approach for structural RNA is that automatic free-energy folding algorithms (UNAFold, mfold, RNAStructure, ViennaRNA) may ignore secondary structure constraints if there is too much conflict with free-energy structures. As a result inferring individual secondary structures for smaller regions followed by a manual quality check should be done (a time consuming task). Secondary structure models are available for a large set of almost complete SSU and fragments of LSU in the SILVA database [56] but most variable regions of LSU remain to be inferred individually. We hope that the consensus secondary structure alignments presented herewith stimulate future studies in the area.

Concerning the procedure employed here for incorporating regions of ambiguous alignment (RAAs), a better solution may be achieved by jointly inferring trees and alignments in a Bayesian framework. In this case, the guide tree and cost regime problem is solved through the joint estimation of phylogenetic tree and sequence alignment, something that is implemented in *Bali-Phy* [57]. Despite problems concerning how to model indels, analyses of large data sets are computationally unfeasible for this program (the largest data set analyzed to date contained 117 terminals, aligned sequences with ~200 bp) [58]. Bracketing the RAAs and aligning them independently would save computational time (since most of non-RAA regions are pre-aligned), would allow parallel calculations for each RAA (91 in our dataset), leading to a biologically more realistic, independent estimate for the parameters of the indel model for each RAA and turning Bayesian alignment into a useful and computationally feasible tool for rDNA genes.

### Evaluating previous hypotheses on acariform relationships

The results presented here are in agreement with several insightful previous works but disagree, in several aspects, with current classifications and published molecular phylogenies. Based on morphological characters and *a priori* transformation series, OConnor [12], presented a higher-level phylogeny of Acariformes showing a basal split among Trombidiformes and Sarcoptiformes (including most Endeostigmata). This dichotomic view of Acariformes phylogeny, however, was first proposed by Enzio Reuter in 1909 [59] and is followed in current, broadly accepted classifications [14]. In OConnor's scheme [12], Sarcoptiformes was supported by the toothed rutellum, differentiated prodorsal region, and the loss of solenidia from tarsi IV. The implied character polarity, however, is not congruent with the relationships inferred in this study. The “Endeostigmata”—Alycidae, Nanorchestidae, Nematalycidae, Alicorhagiidae — share the above mentioned characters (the rutellum was thought to be absent in Nematalycidae, but this was recently dismissed [60]) and was recovered as sister group of remaining Acariformes — the Euacariformes. Moreover, the differentiated prodorsal region is present in the Acariformes’ sister group, Solifugae, hence turning this character state into a plesiomorphy.

The problem here is the absence of unequivocal morphological apomorphies supporting either the clade including most Endeostigmata or Euacariformes. We found a single character in support to the Endeostigmata clade: Nematalycidae presents cuticle projections known as palettes, which are oriented so that their edges are perpendicular with respect to the surface annuli, aiding in the grip of the body against the interstitial surface [61]. These palettes have also been found in the Nanorchestidae [62] where their function is uncertain, and some superficially similar structures may be seen in *Bimichaelia* (Alycidae) (SEM pictures in [63]).

The relationships of Sarcoptiformes recovered here (endeostigmatan families Micropsammidae, Terpnacaridae and Oehserchestidae plus Oribatida, including Astigmata) are in general agreement with the morphology-based analysis by Norton [64]. It is interesting to note that most molecular studies [2, 15, 16] did not support these relationships, with *Gehypochthonius* (Parhyposomata) assuming a more basal position. This basal position seems questionable given the presence of opisthonotal glands, a trait shared among certain derived oribatids and the Astigmata. Our topology also allows testing more specific evolutionary hypotheses, such as the position of the enigmatic mite family Pediculochelidae (*Paralycus*). Previously, this family was placed among Prostigmata, Astigmata or Endeostigmata, but our study places it among protoplophoran oribatids (Fig. 5), supporting the hypothesis that its unusual morphology resulted from paedomorphosis as suggested by Norton et al. [65]. This grouping has a very high support, and it shares many unique modifications in rDNA secondary structure.

Trombidiformes, diagnosed by the loss of primary segmentation of the anamorphic segments AN and PA, the reduction to fewer than four pairs of setae on hysterosomal segment C, and fewer than three pairs on segments D and E [12], was recovered in this study with a high support. In contrast, Prostigmata was recovered with low or no support, mainly due to instability of *Labidostomma* with respect to Paratydeidae and *Hybalicus*. Despite this, *Labidostomma* was consistently recovered in a basal position, which is congruent with the absence of secondary tracheal openings, termed neostigmata, in this taxon [66], presence of six pairs of prosomal setae (a simplesiomorphy shared by Sphaerolichida), two pairs of bothridial setae, and chelatae chelicerae bearing two setae on the fixed digit. In previous classifications, Labidostommatidae was included in Eupodina [11, 13], but its basal position was already recognized in recent classifications [67]. The same simple condition of the stigmata is known for Rhagidiidae, another possible basal taxon (not included in this analysis), while in derived Prostigmata, the stigmata can be either subcheliceral or dorsal (neostigmata).

In our analyses, Eleutherengona was recovered monophyletic, but Eupodina and Anystina were not. It is not surprising since Eleutherengona is a clade supported by many morphological apomorphies: the loss of the third nymphal stage in most taxa; the presence of a sclerotized aedagus in males; the cheliceral bases contiguous or fused, with loss of independent movement; the chelicerae with the movable digit pointed and partly retractable; the genital and anal openings adjacent; a correlated loss of the epimeral organs and genital acetabula; and the fusion of the femoral segments [13, 68]. In this study, however, sampling was limited to Raphignathae, leaving out the Heterostigmata, a clade hypothesized to be the sister group to Raphignathae [13, 68].

A key group in interpreting the non-monophyly of Eupodina and Anystina are the marine mites (family Halacaridae). Traditionally, they were regarded as closely related to the terrestrial predacious superfamily Bdelloidea (=Bdellidae + Cunaxidae) in Eupodina, but we found them to be the sister-group to Parasitengona (includes chiggers and fresh water mites). Atfirst glance, this appears as a major conflict among morphological and molecular data, however, now it is apparent that the traditional classification relies on superficial resemblance rather than accurate consideration of morphology. The position of Halacaridae as Parasitengona's sister group was anticipated by Witte [69] who found six synapomorphies for this relationship: (1) the palp with conspicuous spiniform setae (in most Halacaridae), which are probably homologous to the tibial spine that opposes to the palptarsus and forms the “thumb-claw” process in many Anystina and Eleutherengona; (2) the fixed digit of the chelicerae is reduced, and the movable digit is often hook-like and serrate; (3) sigmoid pieces are sclerotized structures projecting ventrally into the infracapitulum and bend anteriorly, continuing under the capitular saddle as a sclerotized supporting bar, serving as a stabilizing and protecting element for the neostigmatal processes during movement of the cheliceral bases [70]; despite the absence of peritremes in Halacaridae, the sigmoid pieces are present and the chelicerae extend posteriorly beyond them, a similar condition to Parasitengona; (4) the sigmoid pieces extend their proximal portions interiorly, and cheliceral protractor 1 originates on them; (5) a similar mechanism of chelicerae protraction, through forward rotation of the tip of the sigmoid piece; (6) the internal podocephalic canal, although the reduction of the podocephalic glands observed in Halacaridae downplays this character state.

Similarly, several other traditional groupings could not be confirmed in our analyses, suggesting that the current view on the phylogenetic relationships of Prostigmata needs to be revised. Neither Bdelloidea nor Anystidae were recovered. The AU test rejects the hypothesis of a monophyletic Bdelloidea in the three-partition analyses. Morphological support for this placement, however, needs to be investigated more closely in the future. Both Bdellidae and Anystidae have especially conflicting regions of the phylogeny as indicated by the consensus networks (Fig. 3) and IC and ICA values (Additional file 4).

In disagreement with currently accepted classification, but similarly to our results, Otto [71], recovered Anystidae as a non-monophyletic group. In fact, the hypothesis of a close relationship of Anystinae and Erythracarinae was based mainly on the overall appearance — `spider-like' and `long legged, fast and mostly reddish'. Additional characters were: legs arranged in a radiating way, idiosoma lacking a sejugal furrow, movable digit hook-like, presence of 1–3 claw-like setae on the palp tibia and soft cuticle. Since most of these traits were convincingly dismissed as shared apomorphies in a previously published work [71], which is supported by our molecular evidence, we propose to remove the subfamily Erythracarinae from Anystidae and consider it again as a separate family, Erythracaridae Oudemans, 1936 stat. ressur., as it already was used in prior literature [72] (zoobank.org:act:041D4A94-6A5F-4D94-A018-A2C176E6BB1F).

### Likelihood mapping, consensus networks and certainty indices as techniques for phylogenetic signal assessment

Many phylogenetic studies rely solely on Bayesian posterior probabilities and bootstrap proportions to give some indication on how reliable are the inferences, despite other methods of estimation of the robustness of phylogenetic signal that are available. In this study, Likelihood Mapping, Consensus networks on bootstrap trees and the *Internode Certainty* (IC) and *Internode Certainty All* (ICA) indices gave interesting insights on the strength of the data and hence, on the reliability of the analyses.

First, Likelihood Mapping was shown to be very sensitive to systematic errors due to Long Branch Attraction (LBA): exclusion of fast evolving lineages resulted in a larger proportion of quartets resolved according to the recovered topology in all tested groupings, except for Pedipalpi (Fig. 2c). This sensitivity considerably reduces the utility of quartet mapping to test data suitability for phylogenetic analyses, since an alignment may be able to resolve most quartets due to LBA. In our analyses this seems to be the case for the loop partition that has similar percentages of star-like and partially resolved quartets as the stem partition (Fig. 2b), but when the loop bipartitions are plotted in the consensus networks (Fig. 3) it is clear that there is more discordance. Despite these limitations, Likelihood Mapping, jointly with consensus networks, can be useful for LBA detection.

Measures of nodal certainty (IC and ICA) were used to detect the presence of conflicting phylogenetic signals (as opposed to uncertainty or weak signal) in specific nodes of our phylogeny (Additional file 4). Low values of these indices indicate the presence of a conflicting signal for Myriapoda, Pancrustacea, Chelicerata (except for Tetrapulmonata and Solifugae), and basal portions of “Endeostigmata”, Sarcoptiformes, and Trombidiformes. Interestingly, for the grouping Solifugae and Acariformes, the certainty indices are either a unit (all partitions) or approach a unit (stem and loop regions), indicating the absence of conflict for this node.

## Conclusions

Our analyses show that ribosomal genes unequivocally support a sister group relationship between Solifugae and Acariformes (Poecilophysidea). In contrast to existing morphological hypotheses, we found that most Endeostigmata (rare deep-soil mites known from the Devonian) represent a major basal divergence that occurred prior to the split between the two hyperdiverse acariform lineages, Sarcoptiformes and Trombidiformes. Morphology suggests a basal split between Sarcoptiformes and Trombidiformes and places endeostigmatans at the root of Sarcoptiformes. Thus, our findings may have a substantial impact on higher-level classification of acariform mites and indicate that using endeostigmatans may greatly improve the accuracy of time estimations on fossil-calibrated phylogenies. We inferred Astigmata (an unranked hyperdiverse group including many medically and economically important species) as the sister group of desmonomatan oribatids, which is consistent with some, but not all morphological and molecular hypotheses. For a long time the Astigmata was treated separately from oribatids, following the idea of the influential mite morphologist, François Grandjean, who suggested independent origins of opisthonotal glands in Astigmata and derived oribatids [73, 74]. Interestingly, a topology similar to Grandjean's idea was recovered previously based on 18S rDNA and EF1-α sequences [21]. Here we show that this grouping may be due to non-phylogenetic signal present in a single rDNA partition.

Trombidiformes (Sphaerolichida + Prostigmata) was recovered with a high support. Previously Sphaerolichida was treated within the endeostigmatan lineages [75], but later they were moved to Trombidiformes based on the absence of the rutella, primary segmentation, anamorphic segments AN and PA, and the presence of fewer than four pairs of setae on segment C and fewer than three pairs on segments D and E [12]. This was a revolutionary idea at this time, and our analysis strongly supports this grouping. Labidostommatina was recovered as a basal Prostigmata. Among other major lineages of Prostigmata, only Eleutherengona and Parasitengona were recovered. Marine mites (Halacaridae), a globally distributed group with controversial phylogenetic affinities, were recovered as the sister group of Parasitengona, with high support. This is in agreement with a previous detailed morphological study [69], indicating that the current view of placing marine mites close to the terrestrial predacious Bdelloidea should be abandoned and the superfamily Halacaroidea moved to the infraorder Anystina. Our results and previously published morphological data indicate the need for major rearrangement in the family Anystidae (large, fast moving mites): Erythracarinae is elevated to family rank, Erythracaridae stat. ressur., and treated separately from the Anystidae.

Molecular evidence presented in this paper calls for further investigation of possible shared apomorphies of the early derivative Endeostigmata clade (Alycidae, Nanorchestidae, Nematalycidae, Alicorhagiidae) and the clade comprising the remaining Acariformes; including a greater gene and taxon sampling for clades traditionally placed in Eupodina and Anystina (Trombidiformes) and Palaeosomata (Sarcoptiformes), since consensus networks and internode certainty measures (IC, ICA) show that these tree regions are especially conflicting. Finally, our results demonstrate that regions of ambiguous sequence-similarity alignment, when aligned using secondary structure information, may provide useful phylogenetic signal.

## Methods

### Taxonomic sampling and sequencing

We sequenced the small and large subunit nuclear rDNA genes for a total of 118 taxa. Another 110 sequences were retrieved from GenBank. GenBank accession numbers are listed in Additional file 1 along with details on vouchering. The most distant out-group was Priapulida (2 spp). Distant outgroups included all main panarthropod lineages: 2 Onycophora, 32 Hexapoda, 25 Crustacea and 12 Myriapoda. The Chelicerata ingroup comprised 150 taxa (newly sequenced taxa are indicated in parenthesis): 5 Pantopoda, 2 Xiphosura, 9 (3) Opiliones, 24 (21) Araneae, 4 (2) Amblypygi, 3 (2) Uropygi, 7 (5) Scorpiones, 2 (2) Palpigradi, 2 (1) Ricinulei, 2 (1) Solifugae, 6 (4) Pseudoscorpiones, 16 (16) Parasitiformes, and 73 (60) Acariformes.

Molecular work was conducted at the Universidade de São Paulo by ARP following protocols described in [4] and published primers for SSU [76, 77] and for LSU in [78–82] and at the University of Michigan by PBK using previously described protocols and primers [83].

### Ribosomal DNA and secondary structure alignment

Due to its ease of amplification and its among-region evolutionary rate heterogeneity, ribosomal DNA enjoyed a pioneering role in molecular phylogenetics, leading to the so-called “new animal phylogeny” [84, 85], and being – even in the genomic era – the sole source of molecular data for several large clades (*e.g*., [6]).

Yet serious difficulties exist in proposing reliable hypotheses of nucleotide homology for regions of ambiguous alignment (RAAs) using standard multiple sequence alignment procedures. Automated alignment based on cost regimes and maximizing sequence identity proved to be a failure in this respect, mainly due the among-region evolutionary rates of substitution and the incidence of insertions and deletions (indels) [47]. Congruence-based shortcuts, like direct optimization implemented in POY [86, 87], add the artifact of over-optimization or epistemological character non-independence [88, 4].

By providing a causal framework for proposing molecular homology, secondary structure consensus guided alignment contrasts with the methods that rely on simple sequence similarity or congruence optimization. We refer to this framework as ‘causal’ to highlight: (i) that it considers as the main evidence for homology the compensatory changes driven by stabilizing natural selection on secondary structure; (ii) the way in which it contrasts with methods that do not assume any cause for among-sequence divergence and rely only on identity (inheritance); and (iii) the differences from an agnostic standpoint on evolutionary process, as embodied by the congruence methodologies.

Employing secondary structure for aligning sequences has, however, at least two potential drawbacks. First, since automated methods, like *RNASalsa* [89], remain largely underappreciated, secondary structure guided alignment still relies on extremely time-consuming manual alignment. Second, consensus secondary structure alignment cannot be used if there is no common secondary structure among taxa, leaving out some variable regions that may contain useful phylogenetic information.

For the first problem, one cannot offer any easy solution, except for providing a carefully annotated alignment that, alongside other works [17, 34, 90], can give a useful template for adding new sequences. Fasta files containing our alignments labeled with the SSU and LSU rRNA secondary structure are given in Additional file 2. Here, matching parentheses and dots were used to indicate stem and loop regions, respectively; structural helix numbering is given after [91], except for the SSU variable region 4 (V4) for which notations of [92, 93] were used.

Our secondary structure alignment procedure follows [94], except for employing the program *BioEdit 7.2.1* [95] for sequence editing. Reference rRNA structures [4, 34, 96] were used for this alignment. When regions were too variable, potential pairings based on thermodynamics were explored in *mfold* [97] and consensus secondary structures were inferred in *RNAalifold* [98]. Nucleotides in paired regions whose secondary structure was sustained by compensatory mutations across the entire data set were considered as homologous. Regions inferred to be ambiguously aligned were classified into regions of expansion and contraction (REC), non-pairing regions of ambiguous alignment (RAA) and regions of slipped-strand compensation (RSC) [99].

**Fig. 6:**
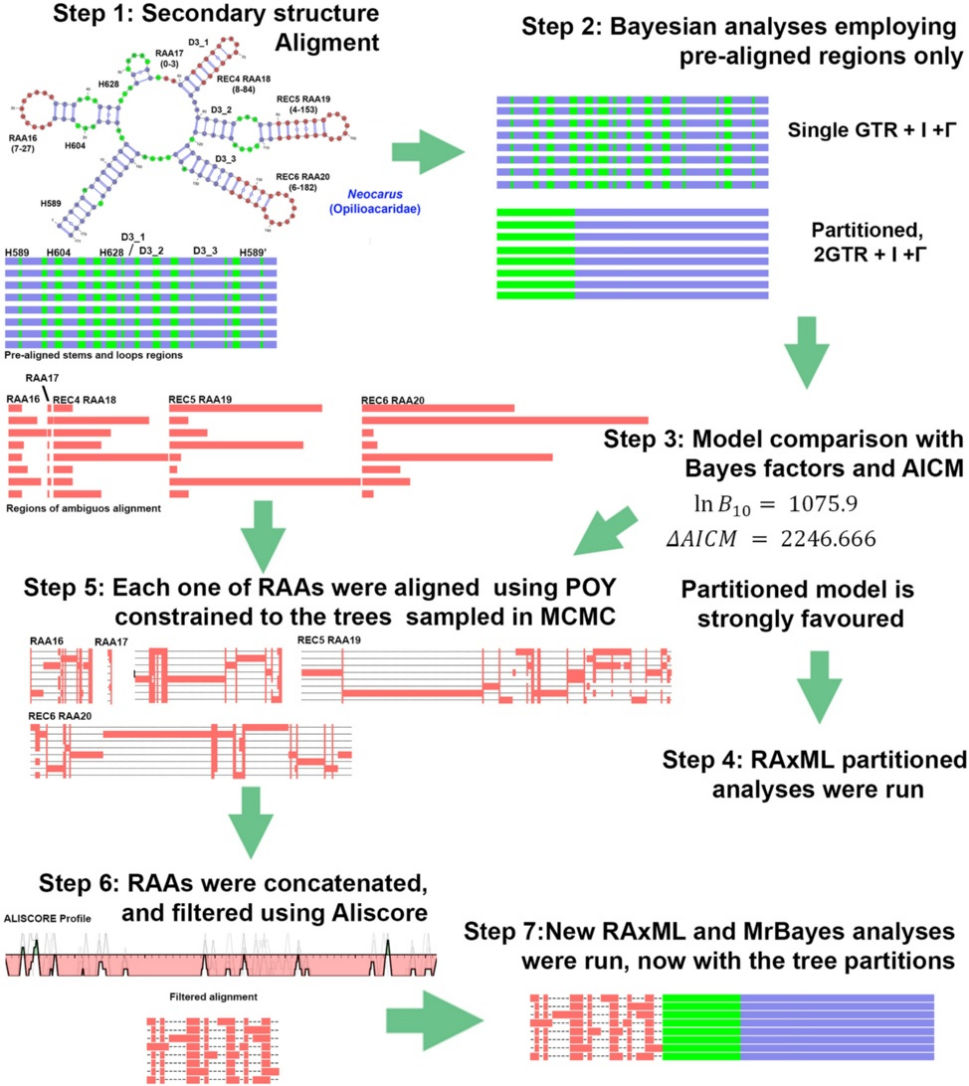
Flowchart of rDNA consensus secondary structure sequence alignment and analyses. Step 1. Multiple alignment guided by secondary structure information detects paired, unpaired and regions of ambiguous alignment (RAAs). Step 2. The RAAs are excluded from the alignment for the first set of Bayesian and ML inferences. Two Bayesian analyses were run, considering either a single model for the concatenated loop and stems or separate substitution models for each of these partitions. Step 3. Bayes factors estimated by Harmonic Mean and AICM strongly favor the partitioned model. Step 4. Partitioned *RAxML* analyses were run. Step 5. Regions of ambiguous alignment (RAAs) were aligned individually using a gap opening cost of four and equally weighted gap extension and substitutions [48] and Markov chain stationary trees from Bayesian analyses as guide trees. Step 6. The resulting concatenated alignment was filtered using *Aliscore*. Step 7. Bayesian and ML analyses employing RAAs alignment as a third partition resulted in topologies largely congruent to those of the two partition analyses, but their nodal support was higher in most cases

We addressed the site homology for RAAs without violating the positional homology inferred for the structural aligned regions. Briefly, regions of ambiguous alignment were initially excluded from preliminary analyses (Step 1–4, flowchart for our methodology on Fig. 6). Then we adapted an existing methodological framework [48] to extract phylogenetic signal from RAAs. Usually, in direct optimization analyses, the choice of weighting values for gaps (opening and extension) and substitutions is driven by maximizing the among-data congruence [100]. When combining conflicting data partitions, this approach can result in permissive gap costs, leading to an artifact where the optimization algorithm overweighs the signal from static partitions relative to those that are dynamically aligned. This phenomenon was discovered independently in two studies and, referred to as ‘over optimization’ [88] or ‘epistemological character non-independence’ [4]. Both studies showed that in order to maintain hypothesis test severity, partitions must be aligned independently prior to concatenation analyses. Liu et al. [48] suggested a cost regime that includes a four-fold gap opening cost relative to equally weighted gap extension and substitution costs and a guide tree as a combination leading to the best nucleotide homology estimates when employing direct optimization. We applied this cost regime for each of the RAAs independently and, instead of a single guide tree, we used MCMC stationary trees sampled from the posterior (Fig. 6, step 5). Then all regions were concatenated, masked in *Aliscore v2.02.2* (with the window size w7: Fig. 6, step 6) and further processed in *Alicut. Aliscore* identifies random similarity within multiple sequence alignments based on Monte Carlo resampling within a sliding window. The method infers similarity profiles from pairwise sequence comparisons and subsequently calculates a consensus profile. Thus, consensus profiles identify dominating patterns of non-random similarity or randomness within sections of multiple sequence alignments. *Alicut* simply slices sites in the alignment according to *Aliscore* output [49, 101]. The concatenated alignment was very long (28,589 nucleotide positions) due a large number of autapomorphic indels. After *Aliscore* filtering, 1,703 positions were included in the analyses (the RAA partition).

Data sets were merged in *FASconCAT v1.0*. This script was also used to read secondary structure mask (in dot-bracket format) and generate a list of pairing and non-pairing positions for downstream phylogenetic programs, like *RAxML* or *MrBayes* [102].

### Model selection and phylogenetic analyses

The GTR + G + I model was selected for all three partitions based on their lowest AIC scores calculated in *jModeltest v. 0.1.1* [103]. We then analyzed secondary structure alignable regions (Fig. 6, step 2) in *MrBayes v.3.2.2* [104], employing four chains, with 2×10^7^ generations, and sample frequency of 1:1000. Convergence was evaluated in *Tracer v.1.6* [105] for continuous parameters; results are given in Additional file 3. Because *MrBayes* default branch length priors and starting tree branch length priors may lead to a gross overestimation of tree length [106, 107], we conducted additional analyses with the starting tree branch length prior set to 0.001 and the prior on the branch length set to 0.01. Results were identical with those run with the default priors. Using stems and loops as two separate partitions improved the harmonic mean of lnL over unpartitioned analysis by 1075.9, and the more accurate AICM estimated using Tracer of 309858.6 (SD = 0.928) for unpartitioned and 307611.9 (SD = 0.738) for partitioned, leading to a ΔAICM of 2246.7 for the unpartitioned analysis, and hence showing a very strong support for the partitioned analyses [108] (Fig. 6, step 3).

For secondary structure informed analyses, we tested 6- and 7-state rDNA-specific models [109] in a Maximum Likelihood context along with the traditional nucleotide (4x4) model in *RAxML 8.1* [110] run on the CIPRES Portal [111]. Employing these biologically sound models, nevertheless, resulted in disappointing outcomes. Letsch & Kjer [22] attributed their also disappointing results, to the poor fitting to loop regions that are subject to saturation. Our tests of saturation in *DAMBE 5.5.2* [19, 20, 112], however, could not detect saturation in any partition (see the Results section above), and an alternative explanation for the poor performance of rRNA models is needed. We suspect it is due to the strong compositional bias toward adenines (see above). Trees inferred with the rDNA-specific models are given in Additional file 4. Topologies discussed in the results sections were based on the simple 4x4 model. For ML analyses 1000 bootstrap replicates were ran.

### Data exploration

The nucleotide composition of our dataset was visualized by the tetrahedric plot function of the R package *Compositions* [113]. The program *TREE*-*PUZZLE 5.2* [114] was employed to explore whether the base composition of each sequence was identical to the average base composition (chi-squared test at a 5 % level).

For comparing rates of molecular evolution, a Bayesian Relative Rates test [115] was conducted using a custom *R* script (Additional file 5).

Two graphical approaches were employed to explore the phylogenetic signal in each data partition and combined datasets, the Likelihood Mapping (LM) [116] as implemented in *TREE*-*PUZZLE*, and the consensus networks [117] using trees generated by bootstrap re-sampling in *SplitsTree4 V4.13.1* [118]. A maximum of 1000 bootstrap replicates for the combined dataset and each partition (loops, stems and concatenated RAAs after filtering in *Aliscore*/*Alicut*) were run in *RAxML*, with a threshold proportion of 0.1.

For the LM, we first assessed the GTR parameter values from the above mentioned unpartitioned ML analysis and then ran partitioned analyses without assigning any grouping scheme (to verify the proportions of further resolved quartets and hence the phylogenetic signal for each partition). Then different groupings were employed to verify the proportion of quartets that support different hypotheses.

### Hypothesis testing

Many long-standing hypotheses on chelicerate, and more specifically, mite relationships (Table 1) were evaluated using the phylogenetically explicit Approximately Unbiased (AU) test in *Consel* [119]. To generate input trees for this test, the best tree representing each hypothesis was calculated in *RAxML* using the constraint command. In the case of a negative constraint, not implemented in *RAxML*, we used the best Bayesian tree (*MrBayes*, two independent runs, 2×10^7^ generations each).

### Quantifying incongruence

Although bootstrap proportions were long considered as measures of robustness of phylogenetic signal, they can be extremely misleading in the presence of phylogenetic conflict among different genes. For example, a node with a bootstrap support of 100 % may appear to be well-supported, although there are a large proportion of gene trees that conflict with that node [120]. Similarly, when applied to bootstrap trees, a well-supported node can represent one overwhelmingly prevalent node over an array of different reconstructions (each having a negligible frequency) or a mixture of two conflicting reconstructions (most frequent and less frequent). In the latter case, bootstrap support can be misleading. To account for this situation, Internode Certainty (IC) and Internode Certainty All (ICA) indices were introduced using Information Theory [121]. IC calculates the degree of certainty for an internode by considering the frequency of the bipartition defined by the internode jointly with that of the most prevalent conflicting bipartition. ICA calculates the degree of certainty for a given internode by considering the frequency of the bipartition defined by this internode *versus* all conflicting bipartitions. Internode certainty values near zero indicate the presence of an almost equally supported bipartition that conflicts with the inferred internode, whereas values close to one indicate the absence of conflict. IC and ICA indices were calculated in *RAxML*.

## Availability of supporting data

All the supporting data are included as additional files.

## Additional files

Additional file 1:
**Sampling data and taxonomy.** Systematic classification of terminals and GenBank accession numbers for sequences and collection information for newly sequenced species and their accession numbers in the CT-UFMG (Coleção taxonômica da Universidade Federal de Minas Gerais) and MZSP (Museu de Zoologia da Universidade de São Paulo). (RAR 93 kb) Additional file 2:
**Secondary structure alignments.** A-B: Two FASTA files containing consensus secondary structure alignments of the 18S and 28S genes. Secondary structure notations masks: brackets “()”, “{}”, and “< >“ indicate paired sites; dots “.” indicate unpaired sites, and asterisks “*” indicate regions of ambiguous alignment (RAAs). Notations for stems and variable regions follow [91] and [92, 93] for the SSU variable region 4 (V4). Length variation for each RAAs is also given separately. (RAR 211 kb) Additional file 3:
**Assessing convergence of MCMC analyses.** Plots of LnL of the stationary phase of the Bayesian analyses and ESS values for parameter estimates. (RAR 600 kb) Additional file 4:
**Trees obtained in analyses using different partitions and nucleotide models; Internode certainty measures for 3- and 2-partition phylogenetic analyses.** A-F: Tree topologies for analyses combining stem and loop regions, all data partitions, and assigning 6A and 7A models for stem and GTR + I + G models for loops. Bootstrap proportion values are given for each node. G: For each node, Internode Certainty (IC) and Internode Certainty All (ICA) indices are shown on ML trees inferred from stem and loop (A) or all regions (B). IC is followed by ICA. (RAR 368 kb) Additional file 5:
**R script for the Bayesian Relative Rates test.** This script implements a Bayesian Relative Rates Test [116], *i.e*. sums of the branch lengths from a node ancestral to the node of interest and its tips for postburnin trees sampled from the posterior. It provides an estimate of the mean, 95 % credible interval, maximum and minimum values for each terminal. (R 6 kb)
